# M2 macrophages promote NSCLC metastasis by upregulating CRYAB

**DOI:** 10.1038/s41419-019-1618-x

**Published:** 2019-05-16

**Authors:** Zhe Guo, Jing Song, Junxia Hao, Hui Zhao, Xiaohui Du, Encheng Li, Yanbin Kuang, Fuquan Yang, Wei Wang, Jiong Deng, Qi Wang

**Affiliations:** 1 Department of Respiratory Medicine, The Second Hospital, Dalian Medical University, Dalian, China; 2 Department of Respiratory Medicine, The First Affiliated Hospital of Shantou University Medical College, Shantou, China; 3 Department of Physical Examination Center, The Second Hospital, Dalian Medical University, Dalian, China; 4 Department of Scientific Research Center, The Second Hospital, Dalian Medical University, Dalian, China; 5 Laboratory of Protein and Peptide Pharmaceuticals and Laboratory of Proteomics, Institute of Biophysics, Chinese Academy of Sciences, Beijing, China; 6 Institute of Microelectronics, Peking University, Beijing, China; 7 Translation Medicine Center, Shanghai Chest Hospital, Shanghai Jiao Tong University, Shanghai, China

**Keywords:** Cancer microenvironment, Non-small-cell lung cancer

## Abstract

The mechanism by which tumor-associated macrophages (TAMs) affect cancer progression is not fully understood. This study developed a microfluidic-based co-culture device to mimic the tumor microenvironment to assess TAM effects on invasion and metastasis in NSCLC. The results showed lung carcinoma cells could cause macrophages to show the M2 (a TAM-like) phenotype, and these M2 macrophages promoted lung cancer cell EMT and invasion. Proteomic analysis by the iTRAQ quantitation strategy and GO ontology of the cancer cells indicated that αB-Crystallin (CRYAB) might be involved in this process. Further, we confirmed the role of CRYAB in cancer invasion and metastasis through cell and animal experiments, as well as human cancer tissue assessment. Overall, we demonstrated that M2 macrophages promote malignancy in lung cancer through the EMT by upregulating CRYAB expression and activating the ERK1/2/Fra-1/slug signaling pathway.

## Introduction

Lung carcinoma (LC) represents the commonest reason for cancer death around the world. In China, LC is the top mortality-causing malignancy^1^, mainly due to metastasis.

Besides the host cells contributing to cancer progression, the host microenvironment also plays an important role in cancer proliferation and metastasis, and may constitute a novel target for developing anticancer therapeutics^2^. The tumor microenvironment (TME) comprises cancer cells and stromal cells, including macrophages, endothelial cells, and fibroblasts, and their products, which are extracellular matrix (ECM) components, cytokines and chemokines, growth factors, enzymes, and cell metabolites^3–5^. Tumor-associated macrophages (TAMs) represent the most frequently encountered stromal cells associated with the immune system in the TME and are alternatively activated^4,6^, promoting cancer proliferation, epithelial to mesenchymal transition (EMT), invasion and metastasis, which results in poor patient prognosis^7,8^. Meanwhile, cancer cells activate macrophages and other non-malignant stromal cells, such as fibroblasts and vascular endothelial cells, in the TME to induce malignancy^9,10^. This suggests a positive feedback between tumor cells and TAMs to promote malignancy^11^. However, the detailed mechanisms by which TAMs promote malignancy remain largely unclear.

Traditionally, in vitro studies of the TME and cancer cell invasion and metastasis use transwell assays and animal models. Yet, these systems lack real-time monitoring tools, and directly assessing interactions of cancer cells with TAMs is challenging. Therefore, an efficient and reliable in vitro TME model is needed. To this end, microfluidic chips, because of excellent biological compatibility, flexibility and low cost, are widely used in cancer studies. In addition, it is convenient to control cell growth and stimuli spatially and temporally^12,13^. This indicates that a microfluidic chip may potentially serve as a model to mimic the TME.

Recently, quantitative proteomic approaches have been successful in assessing the changes and quantities of proteins during cancer development. Additionally, they facilitate the identification of tumor related protein markers and help understand the molecular mechanisms of cancer metastasis. Isobaric tags for relative and absolute quantification (iTRAQ)^14^ represents one of the most popular methods for relative quantification among biological samples in MS analysis. It has the advantages of labeling complex samples with high-throughput and quantifying proteins from different specimens simultaneously.

Utilizing the microfluidic chip and proteomic analysis to assess the lung cancer microenvironment, we focused on a specific protein, αB-Crystallin (CRYAB). CRYAB belongs to the superfamily of small heat shock proteins (sHsps), with a role of cytoprotective molecular chaperone. It prevents stress-associated aggregation of degraded proteins, maintaining easily aggregable proteins in niches of non-native, re-foldable intermediates as parts of large, soluble, multimeric structures^15^. CRYAB is expressed in multiple malignancies, including head and neck, breast, liver and renal cell carcinomas, and glioblastomas^16–19^, promoting cell migration and invasion^20^. Huang et al. demonstrated that CRYAB induces EMT and confers resistance to sorafenib in liver cancer via ERK1/2/Fra-1/slug signaling^15^. Moreover, ERK signaling, Fra-1 and slug are regulators of cell motility and frequently upregulated in metastatic cancers^21–24^. ERK1/2/Fra-1/slug signaling may therefore play a role in cancer invasion and metastasis. However, the roles of CRYAB and ERK1/2/Fra-1/slug signaling in the malignancy potential of lung cancer remain undefined, as well as the potential involvement of macrophages in this process. Here, we postulated that M2 macrophages, which are considered to be TAM-like, induce the malignancy potential of lung carcinoma cells by upregulating CRYAB, which results in EMT, via the ERK1/2/Fra-1/slug signaling pathway.

We developed a bionic microfluidic chip to simulate the TME and assess the invasive ability of cancer cells stimulated by macrophages in real-time. By proteomic analysis, we screened several proteins in cancer cells that might be related to cancer invasion and metastasis. Finally, we targeted αB-Crystallin (CRYAB) and confirmed its function in cancer progression through cell and animal experiments, as well as human cancer tissue assessment. These findings suggested CRYAB should be considered a new regulator of lung cancer invasion and metastasis via ERK1/2/Fra-1/slug signaling; combination of the microfluidic chip and proteomics is a robust platform for assessing the lung cancer microenvironment.

## Materials and methods

### Microfluidic chip development

**Fig. 1:**
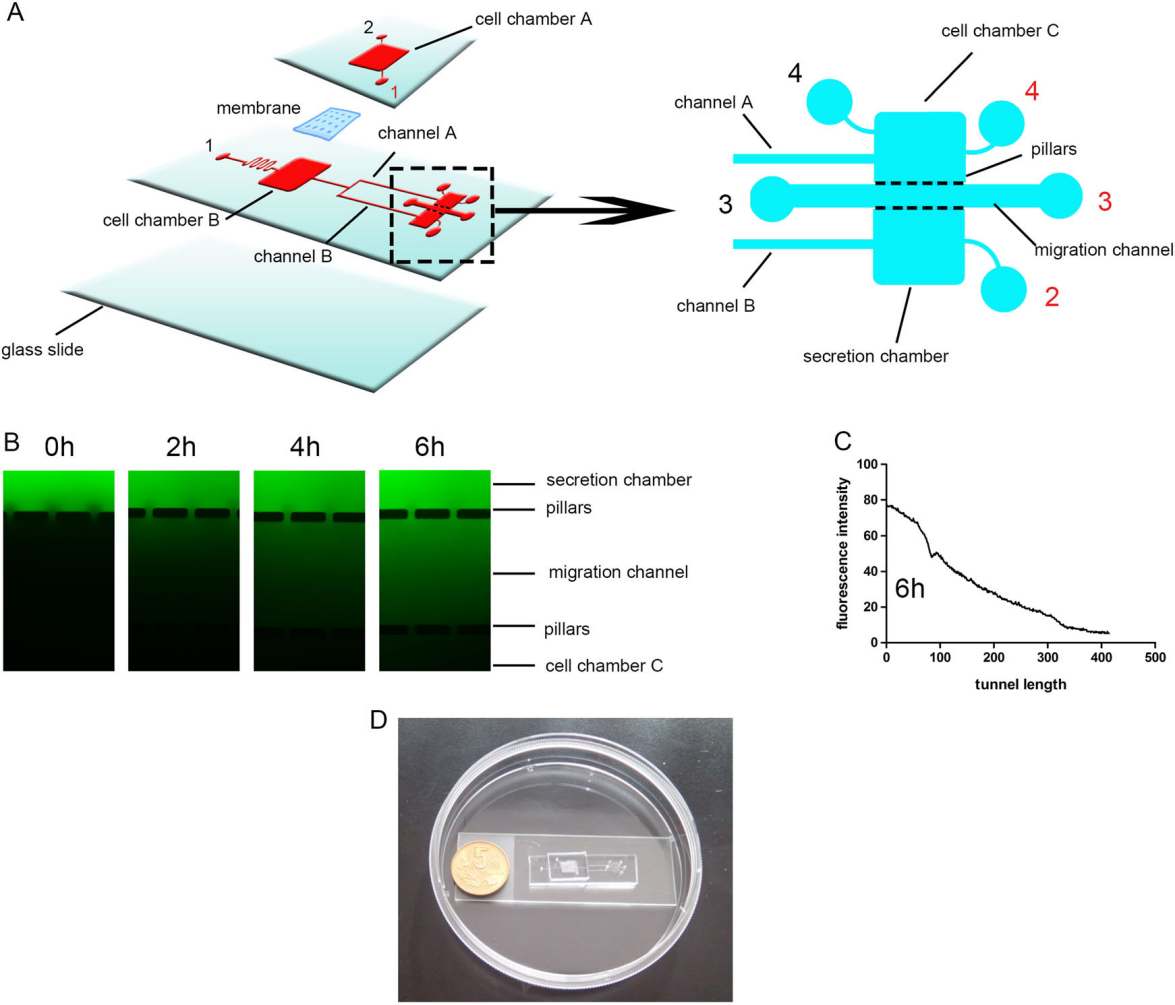
Design and illustration of the integrated microfluidic chip. **a** A schematic diagram of the integrated microfluidic device designed to generate a non-contact cell co-culture model and perform invasion assays. Black and red numbers indicate inlets and outlets, respectively. **b** Representative immunofluorescent images of FITC-dextran forming a concentration gradient across the basement membrane at different time points. **c** Quantitation of immunofluorescence concentration gradients at 6 h in the device. **d** A photograph of the integrated microfluidic device

The microfluidic chip is schematically depicted in Fig. 1a, and was manufactured using Polydimethylsiloxane (PDMS) (Dow Corning, MI, USA) as described previously^25–27^.

The microchip comprised two PDMS layers sandwiching a 0.4-μm pore transwell membrane (Corning) segmented into chambers A and B. The non-small cell lung cancer (NSCLC) A549 and NCI-H1975 cell lines and macrophages were cultured in Chambers A and B, respectively, simulating interactions between cancer and stromal cells, mimicking the in vivo TME. The migration channel (40μm × 500 μm × 2 mm), which was between the two rows of micro-capillaries (gap of 20 μm), could only allow Matrigel (Corning, USA) to flow into the migration channel but not into the adjacent chamber. On micro-channel A and B, two clips served as micro-valves for controlling loading into the subsequent chambers^27^. In this study, NSCLCs were plated into chamber C; after cell adherence, secretion from upstream allows flow into chamber C by shutting down channel B. After stimulation by the upstream secretion for 3 days, A549 cells underwent the EMT, and channel A was shut down and channel B was open; secretion from the upstream channel flowed into the secretion chamber, causing cancer cells to degrade matrigel and advance into the secretion chamber. This chip was designed for cell culture chamber connection with input and syringe pumps connected to individual chambers for controlling culture medium flow from the upper chamber to the lower one.

### Cells and culture

The human NSCLC A549 and NCI-H1975 cell lines, and the mononuclear THP-1 cell line were provided by American Type Culture Collection (ATCC; USA) and cultured in RPMI-1640 (Gibco) containing 10% fetal bovine serum (FBS; Gibco) and an antibiotic cocktail (penicillin and streptomycin at 100 U/mL each) at 37 °C in a humid environment containing 5% CO_2_. Macrophages generated by treatment of THP-1 cells with 150 nM PMA (Sigma) for 24 h were co-cultured with NSCLC cells (1:1) to cause M2 macrophage activation^28^. In return, the NSCLC cells were stimulated to undergo epithelial-mesenchymal transition.

### Immunofluorescence

Immunofluorescence was performed as described previously^27^ with primary antibodies against F4/80 (Santa Cruz), CD206 (BioLegend), E-cadherin (Proteintech, Wuhan, China), N-cadherin (Proteintech, Wuhan, China), Slug (Proteintech, Wuhan, China) and CRYAB (Abcam).

### Immunoblot

Immunoblot was carried out as outlined in a previous report^13^ with primary antibodies against F4/80 (Santa Cruz), CD206 (BioLegend), E-cadherin (Proteintech, Wuhan, China), N-cadherin (Proteintech, Wuhan, China) and Slug (Proteintech, Wuhan, China), CRYAB (Abcam), Phospho-MEK1/2 (Cell Signaling Technology), MEK1/2 (Abcam), Phospho-ERK1/2 (Cell Signaling Technology), ERK1/2 (Cell Signaling Technology), Fra-1 (Abcam) and GAPDH (Proteintech, Wuhan, China).

### Transwell assay

Appropriate Matrigel (Corning) was employed to coat 8-μm pore membranes between the upper and lower transwell chambers overnight at 37 °C. Then, 200 μl of cells (5 × 10^5^/mL) were plated in the upper chamber, with RPMI-1640 and the M2 conditional medium placed in the lower chamber. After incubation (37 °C for 24 h), cells in the upper chamber underwent fixation with 100% methanol (20 min) and Giemsa staining (Solarbio, Beijing, China) for 20 min, and washed with distilled water. Cells on the filter surface were removed with cotton swabs, and those that had passed through the polycarbonate filter were counted at × 100 magnification under a light microscope(Leica, TCSSP5II)

### Quantitative proteomics and data analysis

These experiments were performed as described in supplementary material.

### Transfection with the CRYAB lentivirus expression vector

Wild-type and mutant CRYAB genes were amplified by reverse transcription PCR with cancer cell cDNA samples as templates. Transfection with the CRYAB lentivirus expression vector was carried out as previously described^29^. The plasmid sequences are shown below:

CRYAB-shRNA-top

5′GATCCTGAAAGTCTTGTGACTAGTGCTGAACTCGAGTTCAGCACTAGTCACAAGACTTTCATTTTTGA3′

CRYAB -shRNA-Bot

5′AGCTTCAAAAAATGAAACTCTTGTGACTAGTGCTGAACTCGAGTTCAGCACTAGTCACAAGACTTTCAG3′

Forward 5′GGATCCGATGGACATCGCCATCCACC 3′

Reverse 5′GAATTCCGCTATTTCTTGGGGGCTGCG 3′

### Infection of cells with a GFP-encoding lentiviral vector

Infection with a lentiviral vector harboring the pRNAT-U6.1/Neo plasmid encoding GFP was performed as previously described^30^.

### Tumor formation in nude mouse models

Athymic nude mice (6-weeks-old) were provided by the Laboratory Animal Center of Dalian Medical University. The animals were anesthetized with sodium pentobarbital (50 mg/kg) by intrapleural (i.p.) injection, followed by administration of 0.15 mL of cell suspensions (experimental or control group) into the left side of the chest. A total of 2 to 3 weeks later, tumor growth and metastasis were assessed by in vivo imaging. Photometric analyses of the tumors were performed with the Living Image 3.1.0 software (Caliper Life Sciences). To examine the correlation between fluorescence intensity and tumor size, a caliper was used to measure the dimensions of each xenograft, whose volume (mm^3^) was derived as length × (width)^2^ × 0.4. The animal study had approval from the Laboratory Animals Committee of Dalian Medical University.

### Tissue microarrays and immunohistochemical staining

To assess the association of CRYAB level with lung cancer metastasis, a tissue array containing multiple human lung adenocarcinoma tissue samples (HLug-Ade180Sur-01) was obtained from Shanghai Outdo Biotech (China). The clinicopathological characteristics of the participants and tissue samples are summarized in Table 1. Immunohistochemistry was performed as described previously^31^ with anti-CRYAB primary antibody (Abcam). For each specimen, the total score of CRYAB expression was calculated as staining intensity (negative staining: 0 point; weak staining: 1 point; moderate staining: 2 point; and strong staining: 3 point) multiplied by the point of the percentage of stained cells (positive cells ≤ 25% of the cells: 1 point; 26–50% of the cells: 2 point; 51–75% of the cells: 3 point; ≥ 75% of the cells: 4 point). When the sample was scored ≥ 4 point, we defined it as high expression, and low expression otherwise. The positive control of CRYAB were set up according to lung cancer from the protein atlas website (http://www.proteinatlas.org).

**Table 1:** Correlation between the clinicopathological characteristics and expression of CRYAB in lung cancer

Groups	No.	CRYAB		*P*-value
		+ High Expression	%	
Gender				
Male	49	23	46.9	0.773
Female	41	18	43.9	
Age (years)				
≥60	55	24	43.6	0.647
<60	35	17	48.5	
Tumor diameter (cm)				
>3	58	25	43.1	0.529
≤3	32	16	50	
Tumor differentiation				
I–II	69	33	47.8	0.433
III–IV	21	8	38.1	
Lymph node metastasis				
Yes	49	27	55.1	0.047
No	41	14	34.1	
TNM stage				
I–II	48	16	33.3	0.013
III–IV	42	25	59.5	

### Statistical analysis

Associations of CRYAB level with clinical features were assessed by the chi-square test. Survival analysis was performed by the Kaplan–Meier method. The remaining results were presented as the mean ± SD of three independent tests. Differences among groups were evaluated by one way analysis of variance (ANOVA) with SPSS 19.0 (SPSS, USA) and GraphPad Prism (version 5.0; San Diego, CA, USA). *P* < 0.05 indicated statistical significance.

## Results

### Fabrication of a bionic invasion microfluidic device

This microfluidic device mimicked interactions between NSCLCs and macrophages, and was employed to evaluate tumor cell invasion in vitro (Fig. 1a). The bionic chip was employed to evaluate interactions between A549 or H1975 cells and TAMs after culture in chambers A and B, respectively, for 72 h. Filling the migration channel with matrigel resulted in a stable concentration gradient. FITC was supplemented to RPMI-1640, which progressively diffused into the matrigel and spread to chamber C, forming a stable concentration gradient maintained for more than 6 h in the matrigel^32^. Figure 1b, c shows the concentrations within 6 h and cancer cell invasion for a 48 h period. The medium in chamber C was refreshed to keep the concentration gradient, which allowed the monitoring of cell invasion.

### Transformation of macrophages and NSCLCs on the chip

**Fig. 2:**
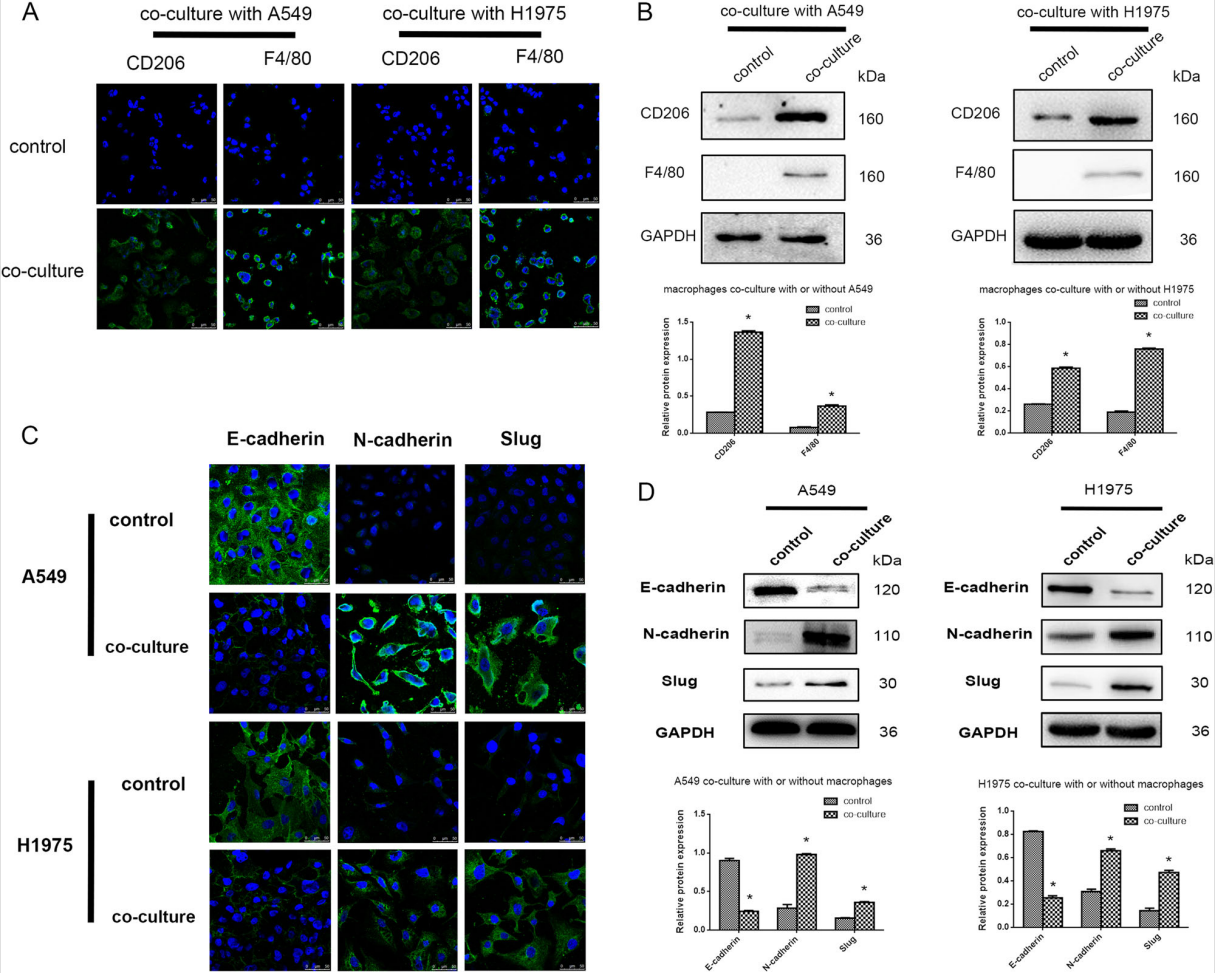
Transformation of macrophages, and A549 and NCI-H1975 cells using the chip and transwell assays. **a** Immunofluorescence detection of CD206 and F4/80 expression in macrophages after co-culture or not with A549 or NCI-H1975 cells in the microfluidic chip for 72 h. **b** Western blot; CD206 and F4/80 expression levels were assessed in macrophages after co-culture or not with A549 or NCI-H1975 cells in a transwell system for 72 h. The quantitative analysis of WB of CD206 and F4/80 in macrophage co-culture with or without A549 and NCI-H1975 cells. **c** Expression of EMT markers in A549 or NCI-H1975 cells with or without co-culture with macrophages for 72 h as assessed by immunofluorescence. **d** Expression of EMT markers in A549 or NCI-H1975 cells with or without co-culture with macrophages for 72 h as assessed by western blot. The quantitative analysis of WB of E-cadherin, N-cadherin, and slug in A549 and NCI-H1975 co-culture with or without macrophage. *P < 0.05 compared to control group

Using this bionic invasion microfluidic device, macrophages were activated into the M2 type after co-culture with A549 or NCI-H1975 cells for 72 h; in turn, A549 and NCI-H1975 cells were caused to undergo epithelial-mesenchymal transition by M2 macrophages after co-culture for 72 h. Immunofluorescence demonstrated that M2 macrophages showed positive CD206 and F4/80 signals unlike non-activated macrophages (Fig. 2a). Co-culture with A549 or NCI-H1975 resulted in overexpression of N-cadherin and Slug, and reduced expression of E-cadherin compared with the control groups; these proteins are related to type 3 EMT markers (Fig. 2c), indicating that these lung cancer cells underwent an invasive or metastatic process. Moreover, these markers were expressed in all cells in transwell systems as assessed by immunoblot (Fig. 2b, d), consistent with the results of the microfluidic chip.

### M2 macrophages induce lung carcinoma cell invasion

**Fig. 3:**
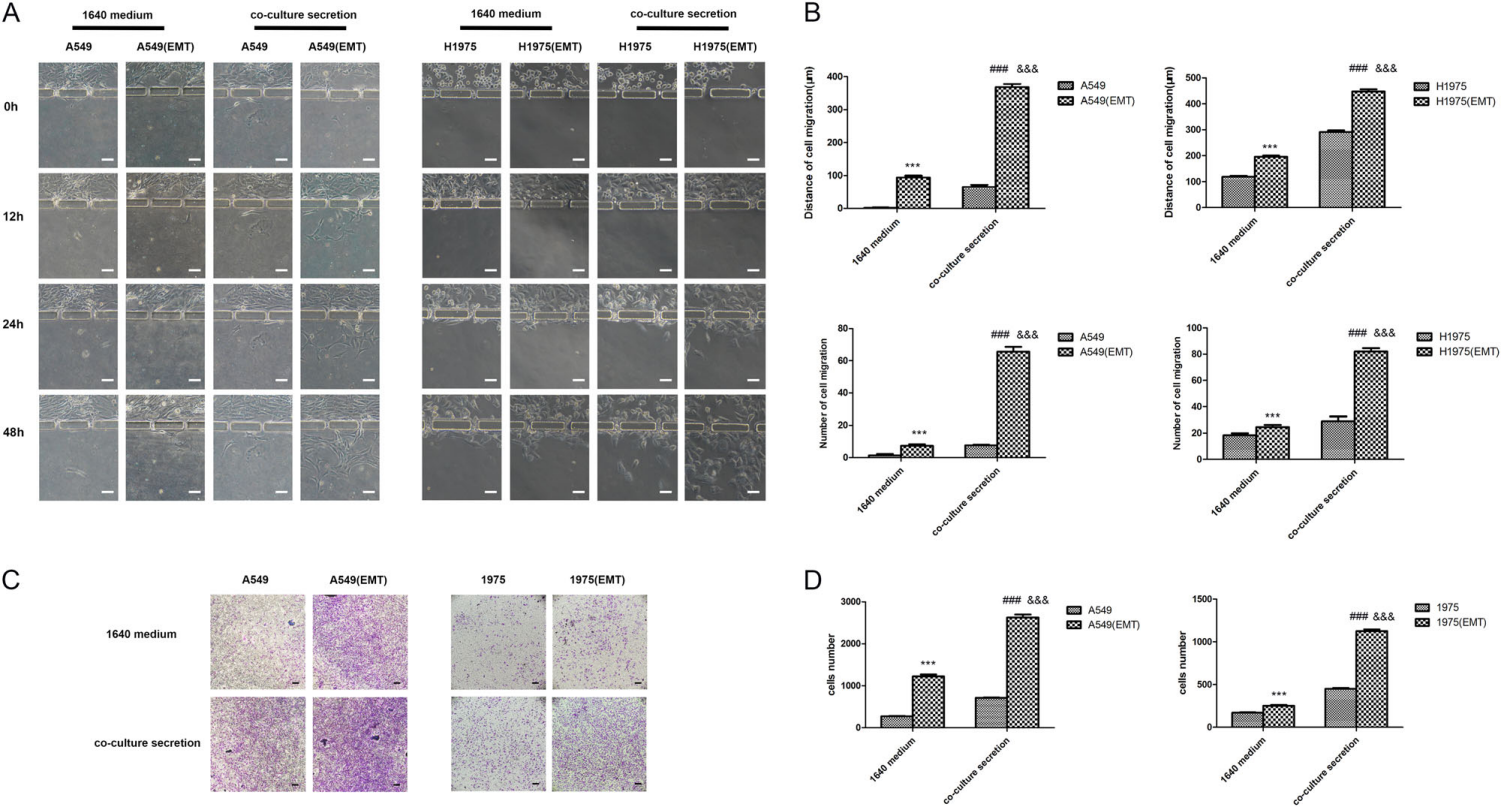
Changes in A549 or NCI-H1975 cell invasion capacity. **a** Representative images depicting the effects of control RPMI-1640 medium and co-culture-conditioned growth media on A549 or NCI-H1975 cell migration at different time points on the chip. **b** Quantitative data showing cell migration distances and the numbers of migrating and invading cells under various treatment conditions at 48 h on the chip. **c** Representative images depicting the effects of control RPMI-1640 medium and co-culture-conditioned growth media on A549 or NCI-H1975 cell migration in transwell assays. **d** Quantitative data showing the numbers of invading A549 or NCI-H1975 cells under different treatment conditions. ***P < 0.001 versus A549 or NCI-H1975 in RPMI-1640 medium; ###*P* < 0.001 versus A549 or NCI-H1975 in co-culture medium; &&&*P* < 0.001 versus A549 (EMT) or NCI-H1975 (EMT) in RPMI-1640 medium. White scale bar, 50 μm. Black scale bar, 100 μm

The downstream of the bionic chip was used to assess the invasive behavior of tumor cells. A549 or NCI-H1975 cells were cultured as control cells, while the EMT A549 or EMT NCI-H1975 cells as test groups in Chamber C. After cell attachment, channel A was closed, while channel B was opened; then, secreted substances from upstream advanced to the secretion chamber. Many activators (1640 medium or co-culture secretion) were placed in the secretion chamber, and cancer cell migration was assessed on an inverted phase contrast microscope for 48 h. After treatment with samples from co-cultures, cancer cells moved towards microchannels with highest amounts of inducers, digesting the matrigel and invading into the secretion chamber^32^. The migratory and invasive behaviors first occurred at 6 h after co-culture secretions were added; however, RPMI-1640 did not show these effects (Fig. 3a). Quantitation revealed cancer cells administered co-culture secretions displayed faster migration. Meanwhile, the EMT A549 and EMT NCI-H1975 groups showed higher invasive cell numbers and longer invasion distances compared with control values (Fig. 3b). Moreover, we confirmed the invasive behavior by the transwell assay (Fig. 3c, d), and the invasive ability of NSCLCs was consistent with microfluidic chip data.

### Differentially expressed proteins as determined by iTRAQ and liquid chromatography–mass spectrometry (LC-MS)/MS

To fully assess protein expression changes, we used iTRAQ labeled 116 and 117 combined with LC-MS/MS. The workflow is shown in Supplementary Fig. 1. In this study, we identified and quantified 5166 proteins from a total of 39,820 peptides in two technical replicates across all 30 runs. The populations fit a Gaussian distribution, and the proteins with ratios lying outside the 95% confidence interval were considered to be significantly differentially expressed. A ratio cutoff >1.491 and <0.674 was applied to quantitative changes to determine upregulated and downregulated proteins. In total, 271 proteins showed significant expression changes, including 155 upregulated and 116 downregulated (Supplementary Table 1).

### Gene ontology findings

A cell undergoes great morphological and phenotypic changes to acquire the invasive capacity through protein expression changes. To obtain a more comprehensive understanding of proteins, cellular components and molecular functions, the 271 above-identified proteins were submitted to clustering by PANTHER. As shown in Supplementary Fig. 2a, b, most of the differentially expressed proteins were enriched in catalytic activity (37.4%) and binding (29.2%). Most of them were located in the cell body (38.7%) and organelles (26.8%). In addition, 3.4% of these proteins were located in cell junctions (e.g., MYO5C, MYO10, SAV1, and PKP3).

Biological process analysis for functional annotation terms (FATs) by DAVID mapped six significant processes directly related to cell invasion and metastasis (*P* < 0.01, FDR < 1%) (Supplementary Fig. 2c and Supplementary Table 2). As examples, cell motion contained 16 proteins (B4GALT1, S100P, PTGS2, TGFBR1, S100A9, ITGA2, DCDC2, ITGB2, EPHB2, PLAUR, CORO1A, ULK1, ITGA5, NCK1, KRT2, and FN1) and cytoskeleton organization comprised 15 proteins (CXCL1, CRYAB, S100A9, TMSB10, MID1, TTN, EPB49, ARHGAP26, KRT9, CORO1A, KRT16, NCK1, KRT14, RALA, and LCP1). Of these proteins, keratins were a conspicuous family, including 13 proteins, which were all significantly upregulated, including keratin1, 2, 5, 6A, 6B, and 14. These molecules participate in multiple processes such as regulation of cell proliferation, cell motion, and cytoskeleton organization. According to previous reports, keratin proteins are involved in cancer metastasis^33,34^. Therefore, proteins related to cell proliferation and motility were further evaluated, and CRYAB was selected as the target marker after preliminary experiments.

### Downregulation of CRYAB inhibits EMT and ERK1/2/Fra-1/slug signaling in lung cancer cells

**Fig. 4:**
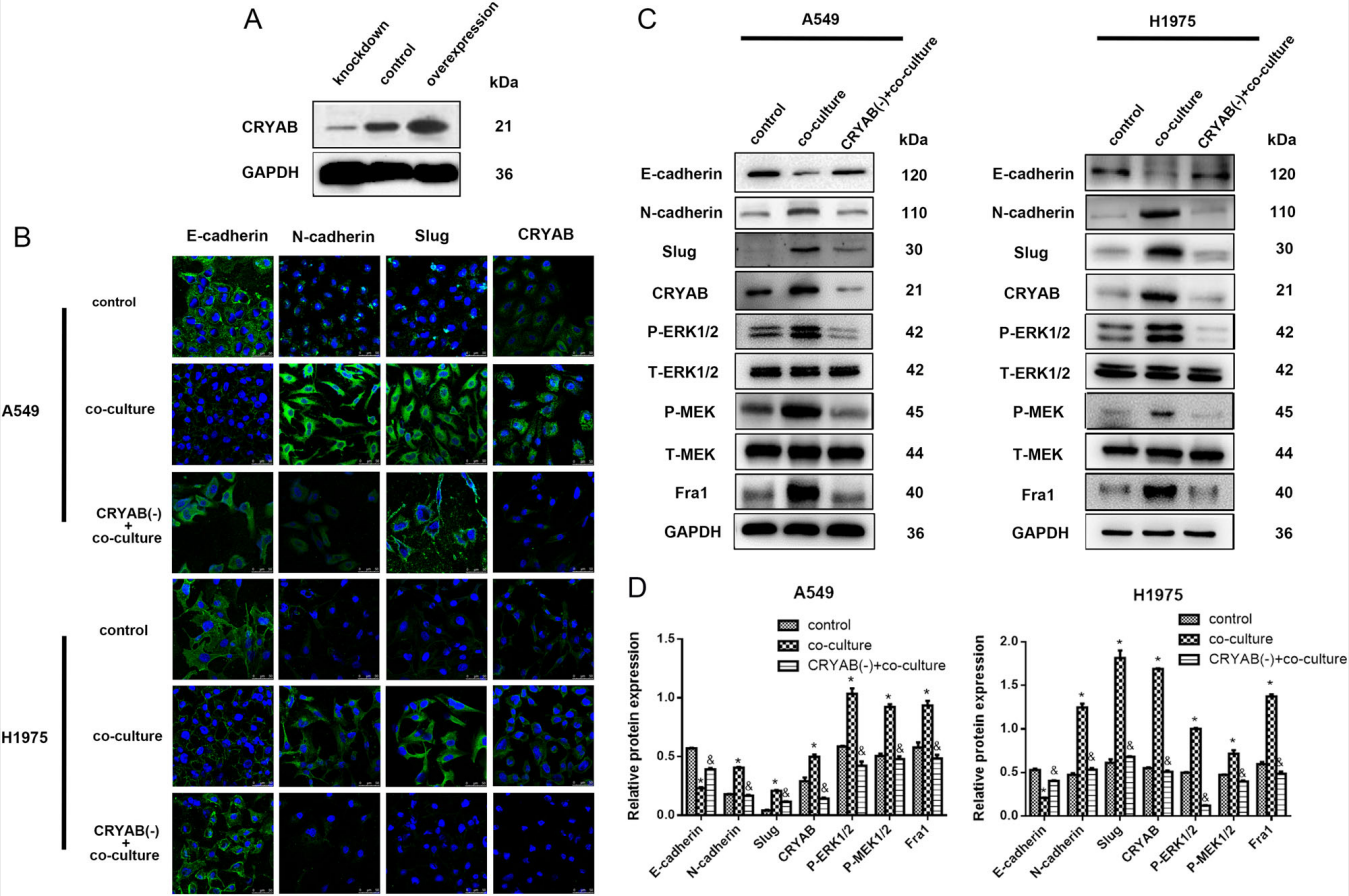
Knockdown of CRYAB decreases EMT in A549 or NCI-H1975 cells after co-culture with macrophages. **a** Verification of CRYAB knockdown and overexpression by western blot. **b** Representative immunofluorescent images for the detection of EMT markers, CRYAB, and ERK1/2/Fra-1/slug signaling pathway proteins in A549 or NCI-H1975 cells in different treatment groups. **c** Western blot showing the expression levels of EMT markers, CRYAB, and ERK1/2/Fra-1/slug signaling pathway proteins in A549 or NCI-H1975 cells in different treatment groups. **d** The quantitative analysis of WB of E-cadherin, N-cadherin, Slug, CRYAB, P-ERK1/2, P-MEK1/2, and Fra in control, co-culture, and CRYAB(-)co-culture in A549 and NCI-H1975 cells. *P < 0.05 compared to control group. &*P* < 0.05 compared to co-culture group

The EMT phenotype, CRYAB, and ERK1/2/Fra-1/slug signaling protein levels were assessed by immunofluorescence and western blot after co-culture with macrophages. Figure 4a depicted successful downregulation and overexpression of CRYAB. Upon co-culture with macrophages, A549 or NCI-H1975 cells with decreased CRYAB levels displayed higher E-cadherin and lower N-cadherin and Slug levels (Fig. 4b, c). Additionally, the above cells showed decreased MEK1/2 and ERK1/2 phosphorylation levels and decreased Fra-1 amounts compared with lung cancer cells of the co-culture group (Fig. 4c). These results indicated that CRYAB was a molecule in tumor cells that might be induced by M2 macrophages; in addition, downregulated CRYAB might decrease EMT and the related ERK1/2/Fra-1/slug signaling pathway in lung cancer. We confirmed the expression of CRYAB and EMT markers in A549 and NCI-H1975 cells in transwell co-cultured systems by western blot (Fig. 4c). As shown above, co-culture secretions of M2 macrophages and A549 or NCI-H1975 cells could induce cancer cell invasion. These co-culture secretions were used as chemotaxin to evaluate the invasive abilities of three different processing models of lung cancer cells (A549 or NCI-H1975, A549, or NCI-H1975 co-cultured with macrophages, and A549 or NCI-H1975 with CRYAB silencing and co-cultured with macrophages).

### CRYAB downregulation reduces invasion in lung carcinoma cells

**Fig. 5:**
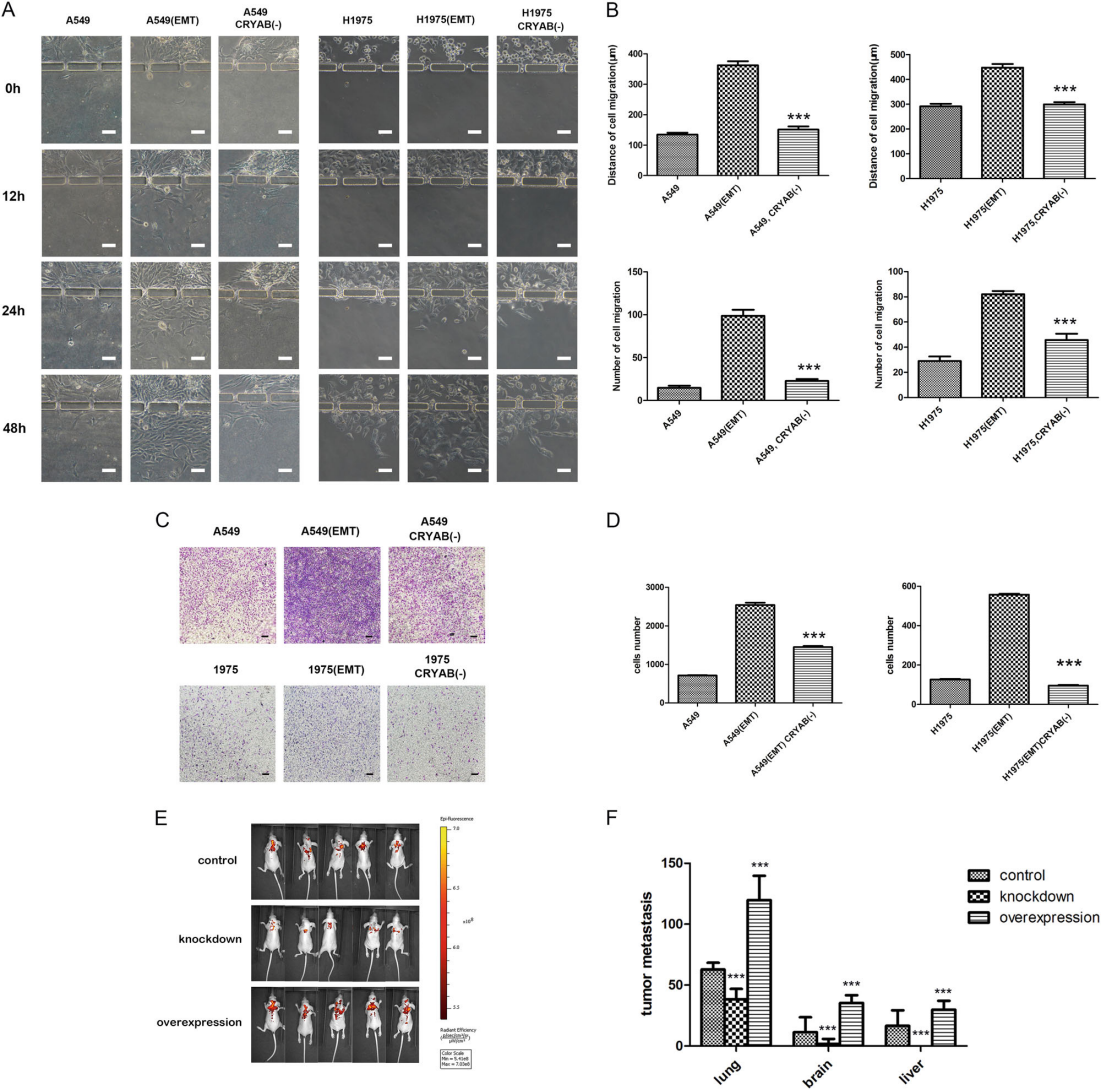
Confirmation of the function of CRYAB in invasion and metastasis. **a** Representative images depicting A549 or NCI-H1975 cell migration in different treatment groups at different time points on the chip. **b** Quantitative data showing cell migration distances and the numbers of migrating and invading cells under different treatment conditions at 48 h on the chip. ***P < 0.001 versus EMT group. **c** Representative images depicting A549 or NCI-H1975 cell migration in different treatment groups using a transwell assay. **d** Quantitative data showing the numbers of invading A549 or NCI-H1975 cells under different treatment conditions using a transwell assay. ***P < 0.001 versus EMT group. **e** Epifluorescence LMB-A549 cells in different treatment groups. **f** Analysis of cell growth in the lung and metastases in the liver or brain in a mouse model. ***P < 0.001 compared to controls. White scale bar, 50 μm. Black scale bar, 100 μm

After knockdown of CRYAB, the number and distance of invasive cells were reduced compared with the values of A549 or NCI-H1975 cells harboring wild-type CRYAB, after co-culture with macrophages (Fig. 5a). The transwell invasive assay confirmed these findings (Fig. 5c). These results indicated that CRYAB downregulation not only decreased EMT, but also reduced invasive ability in cancer cells.

### CRYAB function verification in a mouse model

Verification of CRYAB protein function was performed in a mouse model. Tumor growth and metastasis are depicted in Fig. 5e. There were increased metastatic sites in the lung, liver, and brain after lung cancer cells with CRYAB upregulating were administered; less metastatic sites were found for lung cancer cells with CRYAB downregulation. The results from the bionic chip platform were consistent with those obtained by conventional in vitro and mouse model experiments, indicating that the in vitro chip developed was a reliable platform for assessing the molecular mechanisms involved in tumor biology.

### Associations of clinicopathological characteristics with CRYAB expression in lung cancer

**Fig. 6:**
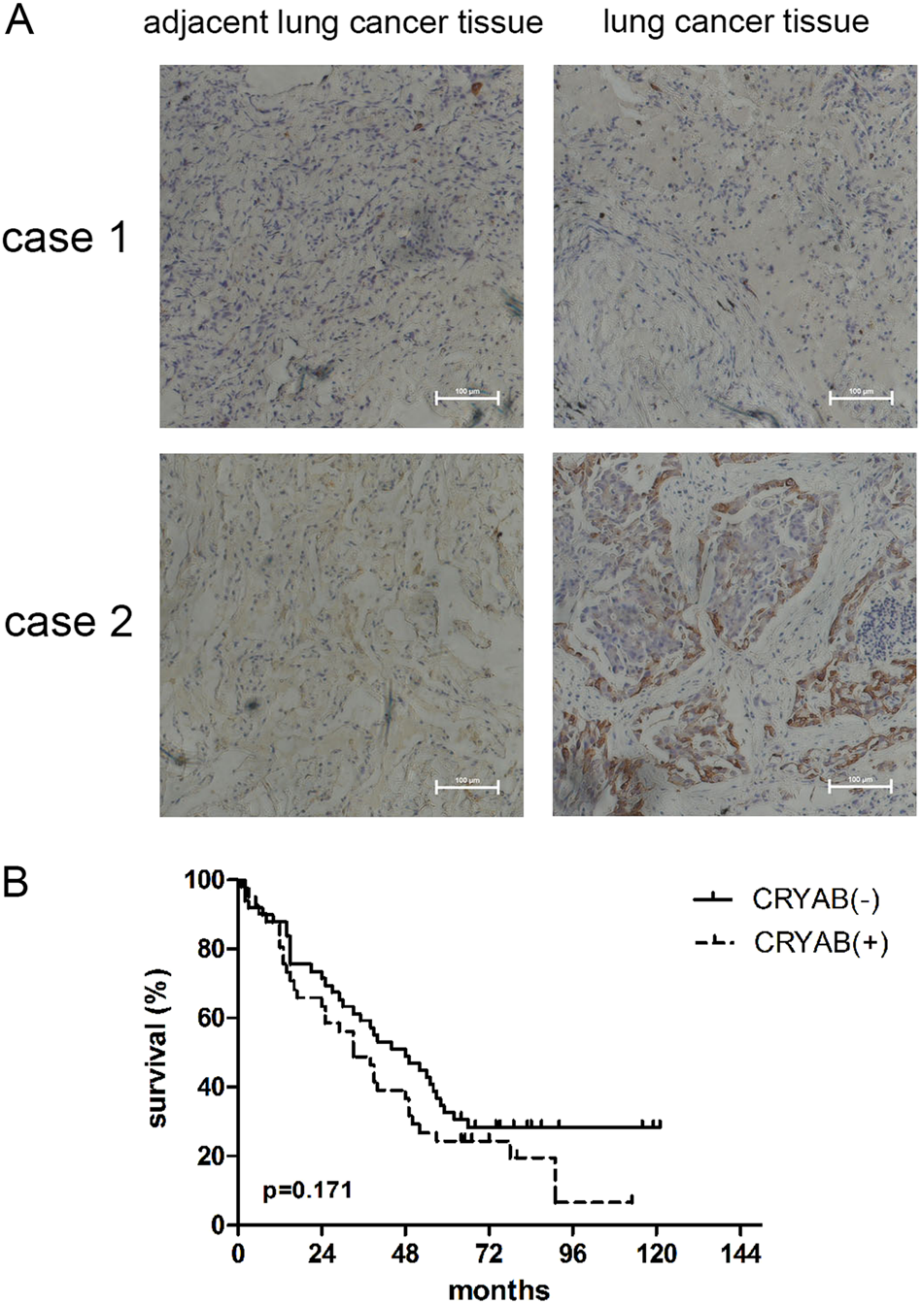
Associations of clinicopathological characteristics with CRYAB expression in lung cancer. **a** Immunostaining of the CRYAB protein in cancerous and adjacent lung tissues from two lung cancer patients. Case 1, no lymph node metastasis; case 2, lymph node metastasis. CRYAB was mainly detected in the cytoplasm of lung cancer tissue from the patient with metastasis, as shown by the brown color. Scale bar, 100 μm. **b** Survival analysis of lung cancer patients by the Kaplan–Meier method. The overall survival rate in patients with high CRYAB protein expression was not significantly lower than that of patients with low or no CRYAB expression

CRYAB expression status was assessed in 90 paraffin-embedded primary lung cancer tissue specimens with matched cancer tissue samples by immunohistochemical staining. As shown in Fig. 6 and Table 1, high CRYAB expression was mainly detected in the cytoplasm of lung cancer samples, showing associations with lymph node metastasis (*P* = 0.047) and TNM stage (*P* = 0.013). Meanwhile, CRYAB expression was not significantly associated with other patient characteristics such as gender, age, tumor diameter, tumor differentiation status, and survival.

## Discussion

Lung carcinoma frequently metastasizes to the bone, brain, and liver, resulting in poor prognosis^35^. Various stromal cells in the tumor microenvironment, including TAMs, directly interact with and are induced by malignant cells, secreting ECM components, as well as multiple growth factors and chemokines to increase the malignancy potential of tumors^36–38^. However, the molecular mechanisms of lung carcinoma invasion and metastasis remain largely undefined, because of the complexity of these processes and the unavailability of reliable and authentic models that can closely mimic the tumor microenvironment in vitro.

This work designed and assessed a bionic microfluidic system, which closely mimicked the in vivo microenvironment of lung carcinoma invasion. With this chip, we successfully co-cultured macrophages and lung carcinoma cells; after 72 h, molecules secreted by lung carcinoma cells activated macrophages to become TAM-like. Meanwhile, these activated macrophages caused cancer cells to undergo EMT. Then, we assessed the role of the TAM-conditioned medium in promoting lung cancer cell invasion; the results showed lung cancer cells had enhanced migratory ability under induction by macrophages. Our findings indicated the following: (i) cancer cells and macrophages formed a positive feedback loop to become EMT and TAM-like, respectively, corroborating findings by Su et al.^11^ in breast cancer; (ii) the conditioned medium obtained from co-culture of M2 macrophages and tumor cells promoted cancer cell invasion, indicating that it is necessary to assess these cells, including cancer and stromal cells, as well as their secretory cytokines simultaneously, which might more closely mimic the in vivo microenvironment, as obtained with the integrated platform provided by our chip; (iii) EMT of tumor cells enhanced their motility, which was simultaneously associated with invasion. Moreover, compared to the platform provided by transwell co-culture systems and the invasion assay, the present integrated bionic chip could control cancer cell proliferation and associated stimuli both in time and space, through precise fluidic flow control. We could also directly assess the distance and number of invasive cancer cells in real-time. These results also suggested the current bionic chip to be a great platform for assessing cell–cell interactions which mimic the in vivo tumor environment.

Furthermore, through assessment of A549 cells by proteomics, immunofluorescence and western bolt, we found CRYAB levels were markedly elevated after co-culture with macrophages compared with cells grown without macrophages. This overexpression was correlated with the invasive and metastatic potential of these malignant cells. Although clinical studies have revealed that CRAYB overexpression in breast cancer, laryngeal squamous cell carcinoma, renal cell cancer and hepatocellular carcinoma is correlated with tumor metastasis and poor prognosis^16–19^, the underlying mechanism is unclear. Huang XY et al.^15^ found that CRYAB leads to increased drug resistance in hepatocellular carcinoma; meanwhile, Malin et al.^19,20^ revealed CRYAB induces breast cancer metastasis to the brain and lungs. However, CRYAB’s role in lung carcinoma, especially its relationship with the tumor microenvironment, remains poorly understood.

This study firstly revealed that M2 macrophages induced invasion in lung cells by upregulating CRYAB. In contrast, after CRYAB silencing, the cancer cells showed attenuated invasion ability. As reported previously, EMT functions to induce cancer invasion and metastasis^39^, and this study revealed that CRYAB silencing noticeably increased the protein levels of E-cadherin, and decreased N-cadherin and Slug amounts in A549 and H1975 cells, suggesting that CRYAB upregulation might occur upstream of the EMT pathway. Moreover, CRYAB upregulation in lung cancer cells by M2 macrophages also induced the ERK1/2/Fra-1/slug pathway, which is related to EMT and likely cell invasion. To confirm these findings, overexpression and silencing of CRYAB in A549 cells were performed, and the resulting cells were tested in mouse models. The results showed that CRYAB upregulation promotes lung cancer metastases to the brain and liver, while down-regulating CRYAB dramatically decreased the metastatic ability, especially to the liver. We also found that CRYAB promoted lung cancer growth in mice. In addition, clinical data showed that CRYAB overexpression in lung cancer was associated with metastasis and a more malignant stage.

This study had limitations. First, M2 macrophages lowly interact with lung cancer cells in the bionic chip culture system, and further stromal cell types, e.g., fibroblasts and endothelial cells, should be incorporated in the future to comprehensively assess the cell–cell interactions taking place. In addition, the sample size of immunohistochemical data was small, and we did not consider treatment modalities, such as radiotherapy and chemotherapy, which might affect patient survival. Therefore, more in-depth clinical studies are required to confirm our findings.

Overall, this study showed that the conditioned medium from co-culture of M2 macrophages and cancer cells promotes cancer cell invasion through EMT and CRYAB upregulation, which in turn induces lung cancer metastasis in vivo. Analysis of lung cancer tissue samples from patients indicated that CRYAB is associated with lymph node metastasis and TNM stage. These findings suggest CRYAB may serve as a novel therapeutic target in lung carcinoma treatment.

## Supplementary information

Supplementary Material

Supplemental Figure 1

Supplemental Figure 2

Supplemental table 1

Supplemental table 2

Supplementary figure legends

## References

[1] Chen W (2016). Cancer statistics in China, 2015. CA Cancer J. Clin..

[2] Shi Y, Du L, Lin L, Wang Y (2017). Tumour-associated mesenchymal stem/stromal cells: emerging therapeutic targets. Nat. Rev. Drug Discov..

[3] Turley SJ, Cremasco V, Astarita JL (2015). Immunological hallmarks of stromal cells in the tumour microenvironment. Nat. Rev. Immunol..

[4] Nagarsheth N, Wicha MS, Zou W (2017). Chemokines in the cancer microenvironment and their relevance in cancer immunotherapy. Nat. Rev. Immunol..

[5] DePalma M, Biziato D, Petrova TV (2017). Microenvironmental regulation of tumour angiogenesis. Nat. Rev. Cancer.

[6] Ngambenjawong C, Gustafson HH, Pun SH (2017). Progress in tumor-associated macrophage (TAM)-targeted therapeutics. Adv. Drug Deliv. Rev..

[7] Chen J (2011). CCL18 from tumor-associated macrophages promotes breast cancer metastasis via PITPNM3. Cancer Cell.

[8] Wei W (2013). FGF18 as a prognostic and therapeutic biomarker in ovarian cancer. J. Clin. Invest..

[9] Joyce JA, Pollard JW (2009). Microenvironmental regulation of metastasis. Nat. Rev. Cancer.

[10] Rolny C (2011). HRG inhibits tumor growth and metastasis by inducing macrophage polarization and vessel normalization through downregulation of PlGF. Cancer Cell.

[11] Su S (2014). A positive feedback loop between mesenchymal-like cancer cells and macrophages is essential to breast cancer metastasis. Cancer Cell.

[12] Xu Z (2013). Application of a microfluidic chip-based 3D co-culture to test drug sensitivity for individualized treatment of lung cancer. Biomaterials.

[13] Li E (2015). Macrophages promote benzopyrene-induced tumor transformation of human bronchial epithelial cells by activation of NF-κB and STAT3 signaling in a bionic airway chip culture and in animal models. Oncotarget.

[14] Ross PL (2004). Multiplexed protein quantitation in Saccharomyces cerevisiae using amine-reactive isobaric tagging reagents. Mol. Cell. Proteomics.

[15] Huang XY (2013). αB-crystallin complexes with 14-3-3ζ to induce epithelial-mesenchymal transition and resistance to sorafenib in hepatocellular carcinoma. Hepatology.

[16] Stegh AH (2008). Bcl2L12-mediated inhibition of effector caspase-3 and caspase-7 via distinct mechanisms in glioblastoma. Proc. Natl Acad. Sci. USA..

[17] Moyano JV (2006). αB-crystallin is a novel oncoprotein that predicts poor clinical outcome in breast cancer. J. Clin. Invest..

[18] Chelouche-Lev D, Kluger HM, Berger AJ, Rimm DL, Price JE (2004). αB-crystallin as a marker of lymph node involvement in breast carcinoma. Cancer.

[19] Malin D (2015). ERK-regulated αB-crystallin induction by matrix detachment inhibits anoikis and promotes lung metastasis in vivo. Oncogene.

[20] Malin D (2014). αB-crystallin: a novel regulator of breast cancer metastasis to the brain. Clin. Cancer Res..

[21] Tanimura S, Takeda K (2017). ERK signalling as a regulator of cell motility. J. Biochem..

[22] Wang L (2018). CCR7 regulates ANO6 to promote migration of pancreatic ductal adenocarcinoma cells via the ERK signaling pathway. Oncol. Lett..

[23] Desmet CJ (2013). Identification of a pharmacologically tractable Fra-1/ADORA2B axis promoting breast cancer metastasis. Proc. Natl Acad. Sci. USA.

[24] Chang JH (2018). Downregulating CD26/DPPIV by apigenin modulates the interplay between Akt and Snail/Slug signaling to restrain metastasis of lung cancer with multiple EGFR statuses. J. Exp. Clin. Cancer Res..

[25] Duffy DC, McDonald JC, Schueller OJ, Whitesides GM (1998). Rapid prototyping of microfluidic systems in poly(dimethylsiloxane). Anal. Chem..

[26] Tilles AW, Baskaran H, Roy P, Yarmush ML (2001). Toner, M. Effects of oxygenation and flow on the viability and function of rat hepatocytes cocultured in a microchannel flat-plate bioreactor. Biotechnol. Bioeng..

[27] Hao Y (2013). Functional investigation of NCI-H460-inducible myofibroblasts on the chemoresistance to VP-16 with a microfluidic 3D co-culture device. PLoS ONE.

[28] Tjiu JW (2009). Tumor-associated macrophage-induced invasion and angiogenesis of human basal cell carcinoma cells by cyclooxygenase-2 induction. J. Invest. Dermatol..

[29] Ishii H (2001). FEZ1/LZTS1 gene at 8p22 suppresses cancer cell growth and regulates mitosis. Proc. Natl Acad. Sci. USA..

[30] Hess KR (2006). Metastatic patterns in adenocarcinoma. Cancer.

[31] Wang Q (2005). Overexpression of endoplasmicreticulum molecular chaperone GRP94 and GRP78 inhuman lung cancer tissues and its significance. Cancer Detect. Prev..

[32] Yu T (2016). Cancer-associated fibroblasts promote non-small cell lung cancer cell invasion by upregulation of glucose-regulated protein 78 (GRP78) expression in an integrated bionic microfluidic device. Oncotarget.

[33] Cheung KJ (2016). Polyclonal breast cancer metastases arise from collective dissemination of keratin 14-expressing tumor cell clusters. Proc. Natl Acad. Sci. USA..

[34] Safadi, R. A., Bader, D. H., Abdullah, N. I. & Sughayer, M. A. Immunohistochemical expression of keratins 6, 7, 8, 14, 16, 18, 19, and MNF-116 pancytokeratin in primary and metastatic melanoma of the head and neck. *Oral Surg. Oral Med. Oral Pathol. Oral Radiol*. **121**, 510–519 (2016). 10.1016/j.oooo.2015.11.016 26906950

[35] Reck M, Heigener DF, Mok T, Soria JC, Rabe KF (2013). Management of non-small-cell lung cancer: recent developments. Lancet.

[36] Wendel, J. R. H., Wang, X. & Hawkins, S. M. The endometriotic tumor microenvironment in ovarian cancer. *Cancers (Basel)* **10**, E261 (2018). 10.3390/cancers10080261 PMC6115869 30087267

[37] DePalma M, Lewis CE (2013). Macrophage regulation of tumor responses to anticancer therapies. Cancer Cell.

[38] Hong, L., Wang, S., Li, W., Wu, D. & Chen, W. Tumor-associated macrophages promote the metastasis of ovarian carcinoma cells by enhancing CXCL16/CXCR6 expression. *Pathol. Res. Pract*. **214**, 1345–1351 (2018). 10.1016/j.prp.2018.07.009 30049511

[39] Campbell K (2018). Contribution of epithelial-mesenchymal transitions to organogenesis and cancer metastasis. Curr. Opin. Cell Biol..

