# Supplementary material for: M2 macrophages promote NSCLC metastasis by upregulating CRYAB
Source: Cell Death Dis. 2019 May 16;10(6):377. doi: 10.1038/s41419-019-1618-x (PMC6522541; doi:10.1038/s41419-019-1618-x)
Supplement: Supplementary file 1 — Supplementary Material [file 41419_2019_1618_MOESM1_ESM.docx]

**Protein Separation, Digestion and Isotopic Labeling**

The cell pellets were washed by ice cold PBS for three times and then suspended in UA (8 M urea in 0.1 M Tris/HCl pH8.5). The lysate was sonicated three times and the remaining debris and unbroken cells were removed by centrifugation at 14,000g at 4°C for 10 min. Protein yield was quantified by BCA assay. Clarified supernatants were transferred to 10 kDa ultrafiltration units and rinsed three times with UA. The total proteins were reduced with 5 mM Tris-(2-carboxyethy) phosphine (TCEP) for 1h at 37°C, followed by blocking cysteine residues in 10 mM methylmethanethiosulfonate (MMTS) for 30 min at room temperature. The filters were washed three times with 50 mM TEAB. Finally, trypsin was added in 300 μL 50 mM TEAB to each filter at a 1:50 trypsin-to-protein mass ratio at 37 °C overnight. The samples were collected at 14,000 g for 10 min. The peptide segment of each sample was labeled using iTRAQ 4-plex kits according to the manufacturer’s instructions (AB Sciex Inc., USA). Digested peptides from A549 and EMT were labeled with 116 and 117 iTRAQ reagents respectively. There constituted reagents were incubated at room temperature for 1 h.

**High-pH RPLC fractionation and Concatenation**

iTRAQ reagents-116 labeled sample and iTRAQ reagents-117 labeled sample were pooled and desalted on an Oasis HLB solid reverse-phase column. The mixture sample was fractionated using a HPLC instrument fitted with an XBridge C18, 250 × 4.6 mm analytical column. The solvent consisted of 2% ACN (pH 10) as mobile phase (A) and 98% ACN (pH 10) as mobile phase B. Briefly, peptides were dissolved in 100 μL buffer A. The gradient of buffer B was set as follows: 5-8% in 5 min, 8-18% in 35 min, 18-32% for 22 min, 32-95% in 2 min，95% in 7 min. Elute was collected every 90s from the fifth minute and totally 45 fractions were obtained. The 45 fractions were concatenated into 15 fractions by merging 1, 16, 31; 2, 17, 32 and dried by vacuum centrifugation.

**Reverse-phase nanoLC-ESI-MS/MS**

Lyophilized samples were resuspended in 0.1% FA and approximately 1- 1.5 μg was brought on column each run. The peptides were loaded onto a 100 μm ID×2 cm fused silica pre-column packed in-house with reverse-phase silica (Reprosil-Pur C18 AQ, 5 μm, Dr. Maisch GmbH, Germany) and then separated on an a 75 μmID×20 cm C18 analytical column packed with reversed phase silica (Reprosil-Pur C18 AQ, 3 μm, Dr. Maisch GmbH). Solvent A was 0.1% FA in water, and solvent B was 0.1% FA in acetonitrile. The gradient used for elution was 5–8% B, 8 min; 8–22% B, 50 min; 22–32% B, 12 min; 32-95% B, 1 min; 95% B, 7 min at a constant flow rate of 280nL/min. The EASY-nLC 1000 HPLC system was online connected to a Q Exactive mass spectrometer (Thermo Scientific). The mass spectrometer was operated in positive ion mode with data-dependent mode. Full scan MS spectra (from m/z 300 to 1600) were acquired in the Orbitrap at a high resolution of 70,000 (m/z 200) with an automatic gain control (AGC) of 3×10^6^. The twenty most intense ions were sequentially isolated and fragmented in the HCD collision cell using 27% collision energy. Fragmentation spectra were acquired in the Orbitrap analyzer with a resolution of 17,500 at m/z 200. A dynamic exclusion time of 40 s was used. All raw data were viewed in Xcalibur v2.2.

**Data Analysis**

For iTRAQ-labeled analysis, the raw data were analyzed with Proteome Discovery v1.4 (Thermo Scientific) using Sequest HT search engine for protein identification and Percolator for FDR (false discovery rate) against a UniProt human protein database (updated on 06-2013). Only tryptic cleavages were considered, with up to two missed cleavages permitted. Database searches were performed with the following parameters: precursor mass tolerance was up to 10 ppm and the product ion mass was up to 0.02 Da. MMTS (C) was set as a fixed modification and iTRAQ 4-plex (N-terminus, K), oxidation (M) were set as variable modifications. Proteins with more than 2 peptides were identified with high confidence.

**GO Ontology Analysis**

Cellular components, molecular functions and biological process for the differential expressed proteins were clustered by PANTHER program [17]. To analyze the specific biological processes of the differentially expressed proteins, the DAVID Bioinformatics Resources (http://david.abcc.ncifcrf.gov/) were used.

**References**

# Thomas PD, Kejariwal A, Campbell MJ et al. PANTHER: a browsable database of gene products organized by biological function, using curated protein family and subfamily classification. Nucleic Acids Res. 2003; 31:334-41.
