# Supplementary figures and images for: M2 macrophages promote NSCLC metastasis by upregulating CRYAB
Source: Cell Death Dis. 2019 May 16;10(6):377. doi: 10.1038/s41419-019-1618-x (PMC6522541; doi:10.1038/s41419-019-1618-x)

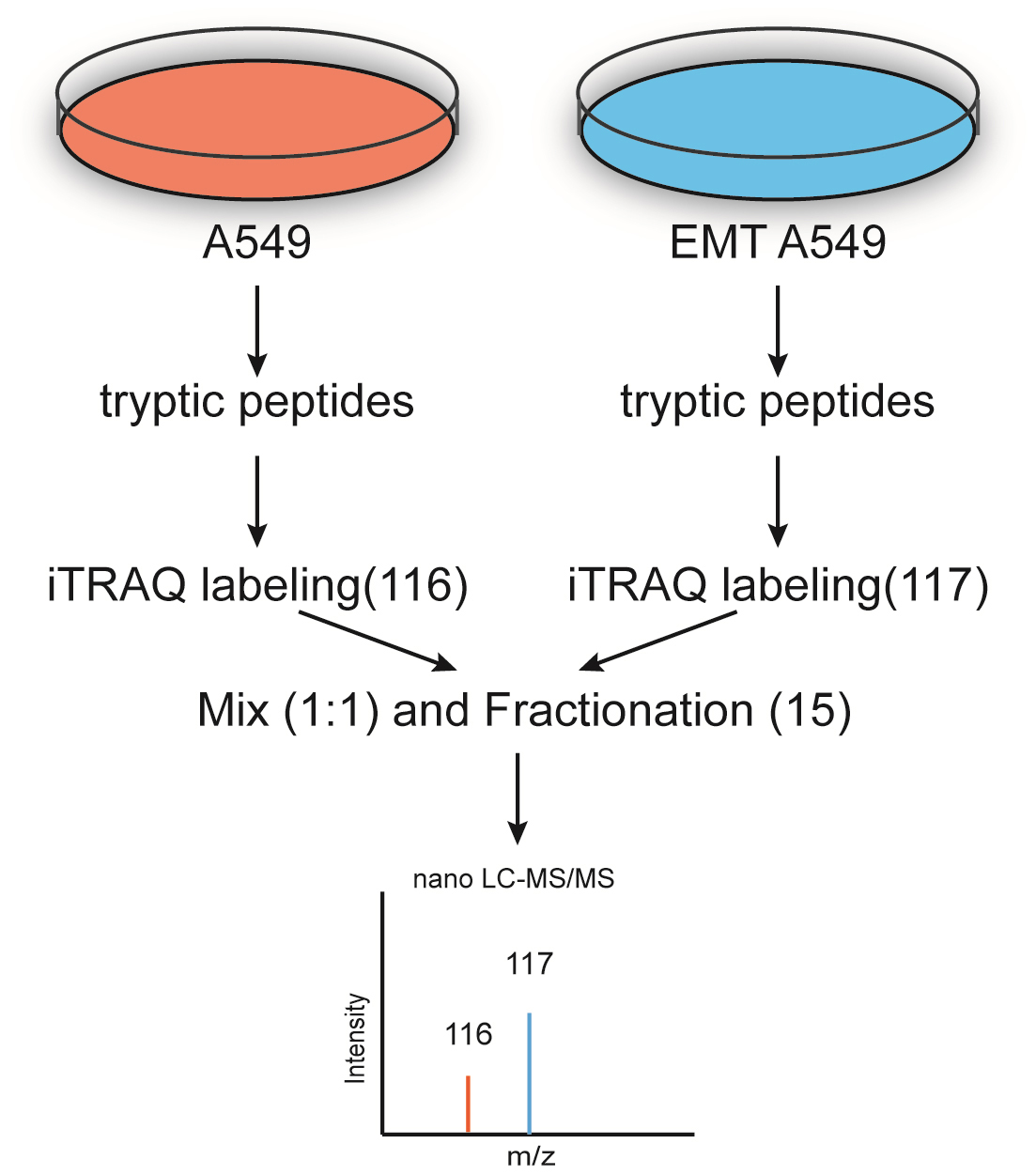

Supplement: Supplementary file 2 — Supplemental Figure 1 [file 41419_2019_1618_MOESM2_ESM.jpg]

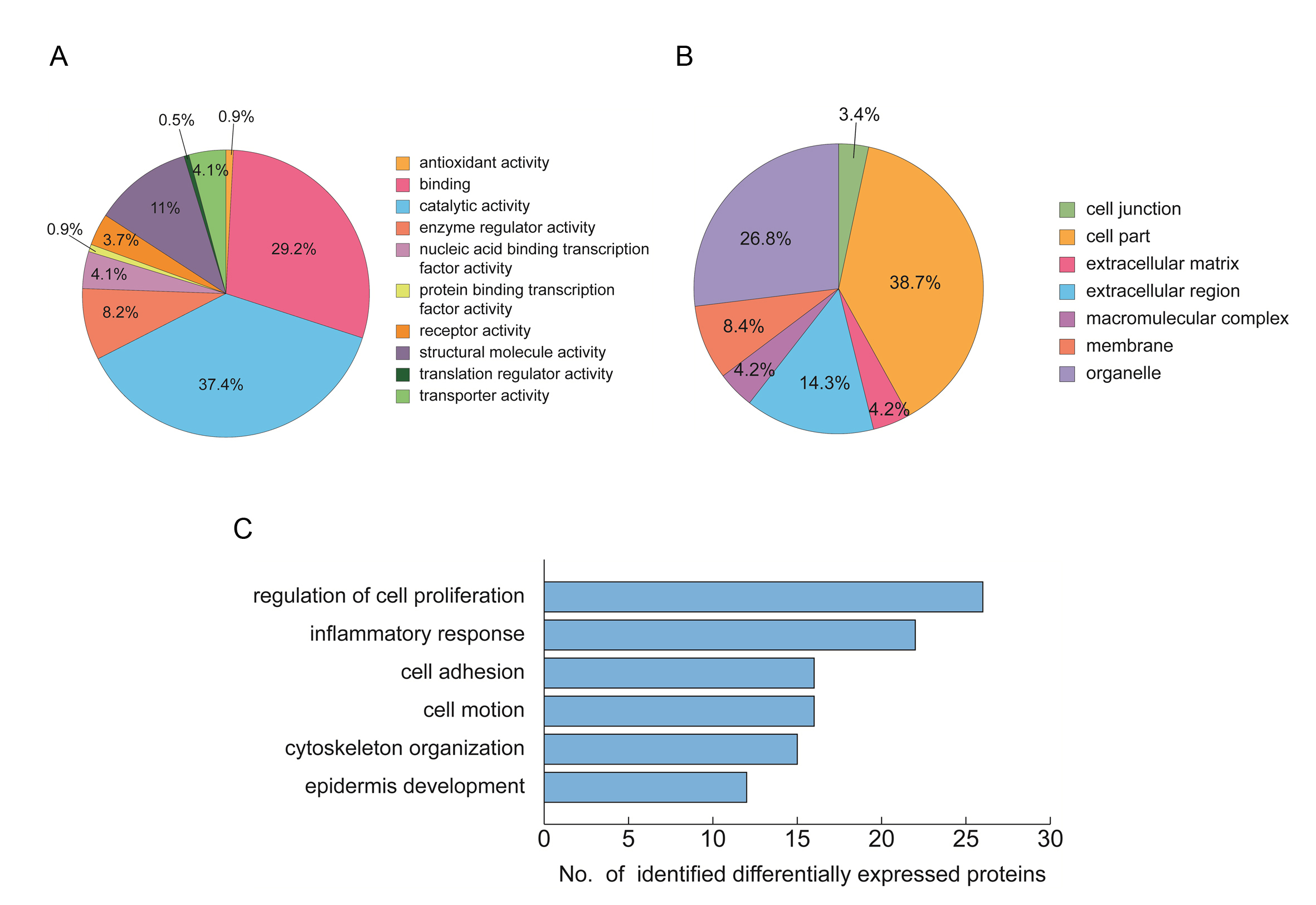

Supplement: Supplementary file 3 — Supplemental Figure 2 [file 41419_2019_1618_MOESM3_ESM.jpg]
